# Editorial: Immuno-diagnosis of active tuberculosis; are we making progress?

**DOI:** 10.3389/fimmu.2023.1092651

**Published:** 2023-02-24

**Authors:** Harriet Mayanja-Kizza, Pere-Joan Cardona, Novel N. Chegou

**Affiliations:** ^1^ Makerere University, Kampala, Uganda; ^2^ Uganda CWRU Research Collaboration, Kampala, Uganda; ^3^ Department of Microbiology, University of Barcelona, Barcelona, Spain; ^4^ Microbiology Department, North Metropolitan Clinical Laboratory, ‘Germans Trias i Pujol’ University Hospital, Badalona, Catalonia, Spain; ^5^ Genetics and Microbiology Department, Universitat Autònoma de Barcelona, Cerdanyola del Vallès, Catalonia, Spain; ^6^ Centro de Investigación Biomédica en Red de Enfermedades Respiratorias (CIBERES), Madrid, Spain; ^7^ DSI-NRF Centre of Excellence for Biomedical Tuberculosis Research South African Medical Research Council Centre for Tuberculosis Research, Cape Town, South Africa; ^8^ Division of Molecular Biology and Human Genetics, Department of Biomedical Sciences, Faculty of Medicine and Health Sciences, Stellenbosch University, Cape Town, South Africa

**Keywords:** tuberculosis, immuno-diagnosis, current trends, potential markers, future directions

Active tuberculosis (ATB) diagnosis suffers from several knowledge gaps and challenges, including the need for newer assays with faster turn-around-time (TAT), high sensitivity and specificity, lower costs, and the potential for Point-of-Care (POC) use assays. Towards this goal, we need novel tests that can be used at the population level to screen and/or test high risk populations, including household contacts of sputum positive index patients and persons living with HIV/AIDS. In addition, tests that can be adaptable towards use in treatment follow-up (to predict cure or relapse/reinfection), and as potential surrogate markers of tuberculosis (TB) vaccination are important towards the global control of tuberculosis. A test using blood-based samples would have potential for future studies to “estimate” the mycobacterial load in the body (akin to the HIV viral load test), which would improve the treatment of ATB, and latent tuberculosis infection (LTBI). In addition, immune-based POC tests for ATB diagnosis have the potential for low invasiveness, being user friendly for staff and patients, minimal training needs, greater simplicity, and low cost; making immuno-diagnosis of active tuberculosis a promising approach. To date, a number of studies have been done in both high and low TB endemic settings to diagnose, or even predict, the evolution towards ATB. Much as some studies have shown high sensitivity and specificity (over 85%), there are still many challenges towards the ideal test, and more research needs to be done.

## Studies for an Immune marker in active tuberculosis diagnostics

Immunodiagnostic studies to diagnose and/or predict the possibility of pulmonary tuberculosis (PTB have recently been conducted in different non sputum specimens, including serum, plasma, unstimulated or stimulated Quantiferon supernatants, peripheral blood mononuclear cells (PBMC), in addition to extra-sanguineous specimens, such as saliva, urine, pleural fluid, ascites, cerebrospinal fluid as well as bronchio-alveolar lavage cells and fluids, and induced sputum. The current literature still shows limited sensitivity and specificity in the diagnosis of ATB, with studies showing variable markers predicting ATB, despite generally related assays and study populations; with few achieving optimal sensitivity and specificity.

Namuganga and colleagues compared immune markers in saliva and sputum among patients with Genexpert confirmed ATB, and showed different significant markers between saliva and serum, thus highlighting the potential for saliva-based ATB diagnostic markers (1). Two multi-country studies in high ATB endemic African sites showed potential of Mtb host response markers in different combinations with varying levels of sensitivity and specificity (up to 94 and 73% respectively) either in predicting ATB compared to healthy controls, (or better still) to Non-MTB respiratory infections (2, 3). However, these studies required 7 and 4 host marker combinations, respectively, to attain the high sensitivity observed, making them expensive to produce for population based ATB triage assays. Moreover, there was little overlap of significant host markers (only CRP and IP-10) in these 2 studies, indicating possible site, technique or individual variation, impacts on the results obtained for particular markers.

In this collection, Luo et al. show that a single candidate marker HLA-DR on Mtb responding specific cells could differentiate ATB from LTBI with a high area under the ROC (receiver operating characteristic) curve, (0.901), and high sensitivity, but moderate specificity, a step towards a low cost potential triage test marker. Furthermore, the same group shows that HLA-DR on Mtb-specific cells, combined with TBAg/PHA ratio increased the ROC to 0.937. However, not all markers are upregulated in ATB, as in the paper by Garlant et al., where in a 6-molecule biosignature, transmembrane protein 49 (TMEM9) was downregulated in ATB compared to LTBI negative controls. In a meta-analysis, Meca et al. suggest that CRP alone, above 8 mg/L has potential as a single molecule triage diagnostic test. Related to this, high sensitivity (hs) CRP alone may differentiate various forms of pediatric ATB compared to healthy children, making this a potentially lower cost, variable age, triage test (4). Another pediatric test by Tornheim et al. suggests plasma kynurenine levels could be useful as an ATB diagnostic marker in children, having high sensitivity (81.5), but low ROC (0.667). It, would, however, be of interest to determine the performance of the combined assessment ofhsCRP and kynurenine levels in pediatric populations. Mann et al. show 4-5 high sensitivity biomarker signatures in patients with confirmed spine ATB relative to control subjects, which may diagnose other forms of extra-pulmonary tuberculosis.

In Brazil, a study by Queiroz et al. of persons with advanced HIV disease indicates the diagnostic potential of an assay with two cytokines, IL-15 and IL-10, in plasmathat are lower in ATB compared to those that received early anti ATB treatment. This emphasizes the possible role of low-level biomarkers in certain disease states, a fact that is often overlooked, or ignored as a “negative result” as increased levels in a biosignature are often expected in diagnostic studies.

Among pregnant women with active tuberculosis, Ranaivomanana et al.’s find lower IGRA responses, probably related to the protective reduction in inflammatory immunity during pregnancy, a finding of relevance to studies in pregnant women.

## Serology tests

Serology has over the years been considered an attractive low cost option for the detection of ATB, with a number of products on the market, and used in low income high endemicity countries, despite most not being validated, or recommended for use in the general population. However, it is thought that some serological tests can be used as adjunctive diagnostic tests in HIV-infected patients with smear-negative PTB or extra-PTB, despite their current limitations (5). Although serum antibodies to ESAT-6 and CFP-10 were considered an attractive option to differentiate between LTBI and ATB, a related study performed in high and low ATB endemic countries, failed to show an ability to differentiate Mtb infection from PTB (6). Another sero-diagnostic study combining four MTB antigens ESAT-6, CFP-10, CFP-21, and MPT-64 did not significantly differentiate PTB from LTBI (7). However, an antibody study in South Africa shows promise when used in combination with cytokine markers (8). Recently, a new promising serological lateral flow assay (LFA) has entered the market (LIODetect^®^TB-ST). This POC test detects IgG, IgA and IgM antibodies against purified recombinant protein antigens PstS1 and PstS3 together with highly purified lipoglycan of Mtb cell wall in serum, plasma or whole blood within 20 min (9). Intrestingly, Nziza et al. show a serological multiplex assay including 7 MTB antigens and 3 novel antibodies that is able to discern LTBI and ATB across HIV positive and negative populations.

## Other potential markers

In a 16-article meta-analysis, the ovarian cancer tumor marker CA-125 has shown promise for detection of ATB with a pooled sensitivity of 0.85 (10). This highlights the importance of thinking outside the box, to identify novel biomarkers and the potential of usingbiomarkers associated with other disease conditions to diagnose ATB.

## Future directions

Overall, there has been extensive research towards an ideal test for immunodiagnosis of ATB. However, there is a need for more research with innovative approaches. These could include further studies looking at cell-mediated responses, with various cytokine/chemokine responses, in different combinations, and the possibility of combining these with antibody responses. In addition, costs should be considered by developing tests with the lowest possible number of biomarkers, as well as considering the availability for POC assays at a population level.

Other options could include blood transcriptomic signatures and blood immune-profiling, immune metabolic markers (e.g. endocrine markers, and pro-inflammatory markers), the use of novel antigens, or combinations of different antigen stimulation responses. Also, studies using body samples other than blood, like saliva, sputum, exudative fluids, bronco alveolar lavage (BAL), breath condensate, urine, and pleural, ascitic or pericardial fluids, could lead to novel immune markers for ATB diagnosis(pulmonary and extra pulmonary). In addition, different diagnostic approaches, such as LFA-POC tests, and other techniques that are faster and have lower costs, diagnosis lower cost test need to be further investigated.

## Author contributions

All authors listed have made a substantial, direct, and intellectual contribution to the work and approved it for publication.
