# Erratum: Editorial: Immuno-diagnosis of active tuberculosis; are we making progress?

**DOI:** 10.3389/fimmu.2023.1282008

**Published:** 2023-09-01

**Authors:** Frontiers Production Office

**Affiliations:** Frontiers Media SA, Lausanne, Switzerland

**Keywords:** tuberculosis, immuno-diagnosis, current trends, potential markers, future directions

Due to a production error, there was an error in affiliation 7. Instead of “DSI-NRF Centre of Excellence for Biomedical Tuberculosis Research South African Medical Research Council Centre for Tuberculosis Research, South Afriacan, South Africa”, it should be “DSI-NRF Centre of Excellence for Biomedical Tuberculosis Research South African Medical Research Council Centre for Tuberculosis Research, Cape Town, South Africa”.

The publisher apologizes for this mistake.

The original version of this article has been updated.

